# Influence of INF-gamma gene polymorphism in HTLV-1 proviral load

**DOI:** 10.1186/1742-4690-8-S1-A119

**Published:** 2011-06-06

**Authors:** Rodrigo Haddad, Maurício C Rocha–Junior, Daiani C Cilião-Alves, Virgínia M D Wagatsuma, Oswaldo M Takayanagui, Eduardo A Donadi, Dimas T Covas, Simone Kashima

**Affiliations:** 1 Regional Blood Center of Ribeirão Preto, Ribeirão Preto, Brazil; 2 Faculty of Medicine of Ribeirão Preto, University of São Paulo, Ribeirão Preto, Brazil; 3 Faculty of Pharmaceutical Sciences of Ribeirão Preto, University of São Paulo, Ribeirão Preto, Brazil

## Background

Interferon-gamma (INF-γ) is a key cytokine involved in the defense against intracellular pathogens, such as HTLV-1, by coordinating the expression of immunologically relevant genes. Previous studies showed the importance of this cytokine on HTLV-1-related pathogenesis. Thus, we have investigated the possible association between IFN-γ gene single-nucleotide polymorphism linked to high and low producer phenotypes (IFN-γ [+874T(high) → A(low)]) and risk of development of symptoms related with HTLV-1 infection and proviral load.

## Methods

The polymorphism +874 T/A of INF-γ was analyzed by PCR-SSP in 93 patients HLTV-1 positive (HAC + HAM), stratified according to the presence (HAM, n = 50) or not (HAC, n = 43) of symptoms, and healthy controls (n = 150). Proviral load of infected patients (HAC and HAM) was determined by real-time PCR.

## Results

No significant difference was observed for allelic and genotypic frequencies of the +874T/A polymorphism of INF-γ when correlated with HAC, HAM and healthy controls groups. The median of proviral load was lower in HAC than HAM group (p=0.0131). Also, the p-value is very close to significance (p=0.0523) for +874TT genotype (high producer of INF-γ) and low proviral load, compared to the genotypes +874AA and +874AT.

## Conclusion

Despite the lack of significant associations, the low proviral load appears correlated with high producer of INF-γ (+874TT) genotype. Increasing the number of patients may lead to statistical relationship of the +874TT genotype with low proviral load.

